# *fliC* Mediates *Pseudomonas plecoglossicida*’s Hijack of Inflammatory Immunity and Glucose Metabolism in the Large Yellow Croaker

**DOI:** 10.3390/antiox14101189

**Published:** 2025-09-28

**Authors:** Xizhi Peng, Yujia Sun, Huanjiao Tan, Huanying Pang, Caiyuan Zhao, Qingpi Yan

**Affiliations:** 1 Key Laboratory of Healthy Mariculture for the East China Sea, Ministry of Agriculture, Fisheries College, Jimei University, Xiamen 361021, China; newt@jmu.edu.cn (X.P.); sunyj@jmu.edu.cn (Y.S.); tanhuanj@jmu.edu.cn (H.T.); 2 State Key Laboratory of Mariculture Breeding, Fisheries College, Jimei University, Xiamen 361021, China; 3 Fisheries College, Guangdong Ocean University, Zhanjiang 524025, China; panghy@gdou.edu.cn; 4 Guangdong Provincial Key Laboratory of Aquatic Animal Disease Control and Healthy Culture, Zhanjiang 524025, China; 5 Animal Science and Technology College, Henan University of Animal Husbandry and Economy, Zhengzhou 450046, China; 231029@hnuahe.edu.cn

**Keywords:** *Pseudomonas plecoglossicida*, *fliC*, large yellow croaker, flagella, Tlr5/NF-κB, glucose metabolism

## Abstract

The bacterial flagellum plays a crucial role in pathogenesis. However, the mechanism by which the flagellum interferes with host energy metabolism remains unclear. In this study, we confirmed that deletion of the *fliC* gene resulted in a 30% reduction in the virulence of *Pseudomonas plecoglossicida* against the large yellow croaker (*Larimichthys crocea*). Compared to the wild-type strain (WT) infection group, the Δ*fliC* infection group exhibited a 29.54% decrease in the number of vacuolar degeneration hepatocytes and a 50.83% higher liver glycogen content. Furthermore, infection led to decreased mitochondrial complex V activity, a reduced NAD+/NADH ratio, lower levels of reduced glutathione (GSH), and increased lipid peroxide levels; however, these metabolic perturbations were significantly ameliorated in the Δ*fliC* group compared to the WT group. Proteomic analysis revealed that the dysregulation of the complement cascade and core carbon metabolic pathways observed in the WT infection group liver was significantly alleviated in the Δ*fliC* infection group. Additionally, in the Δ*fliC* infection group, pro-inflammatory genes (such as *Tlr5*, *Tnfα*, and *Il1β*) were downregulated, while lipid metabolism-related genes (such as *Acox1*, *Cpt1a*, and *Pparα*) were upregulated, suggesting the suppression of the Tlr5/NF-κB immune signaling axis and enhanced fatty acid *β*-oxidation. Collectively, *fliC* may mediate the disruption of host glucose and lipid metabolism homeostasis through a Tlr5-triggered immunometabolic regulatory axis. In conclusion, this study demonstrates that bacterial flagella modulate host energy metabolism, expanding our understanding of flagellum-mediated pathogenesis.

## 1. Introduction

The bacterial flagellum is a precision nanomachine assembled through a strictly programmed hierarchical process [1]. As the final extracellular component assembled, the flagellar filament propels bacterial motility, enabling pathogens to colonize host niches efficiently [2]. During host invasion, the flagellum confers versatile functionality upon pathogenic bacteria, enabling remarkable biological adaptations such as sophisticated immune evasion mechanisms [3,4]. As a specialized extracellular structure of bacteria, flagellin is specifically recognized by the host’s TLR5, thereby initiating downstream immune responses including the activation of the NF-κB pathway to induce pro-inflammatory responses [5]. Immune activation and execution are highly energy-intensive processes; therefore, flagella can indirectly disrupt host energy metabolic homeostasis. For example, the flagella of *Escherichia coli* stimulate the host to produce excessive reactive oxygen species (ROS), thereby affecting mitochondrial activity, and the flagella of *Candidatus Berkiella cookevillensis* exploit the glycolytic pathway of amoebae to fuel its proliferation [6,7]. However, the mechanism by which bacterial flagella mediate the dynamic equilibrium between host energy metabolism and immune regulation remains elusive.

*Pseudomonas plecoglossicida* is a Gram-negative, rod-shaped bacterium with polar flagella. It is a pathogen causing childhood pneumonia and fish metabolic dysregulation [8,9]. The deficiency of flagella impaired *P. plecoglossicida*’s swarming motility, chemotaxis, adhesion, and biofilm formation [10]. Dynamic monitoring revealed flagellum-related genes were significantly upregulated post-infection [11]. In addition, the flagellum not only regulated immune pathways such as complement activation and immune effector processes but also interfered with metabolic functions [12]. However, the mechanism by which the *P. plecoglossicida*’s flagellum disrupted the host TCA cycle remains unclear.

This study will construct a *fliC* (ACRRS2_09385)-deficient mutant strain (Δ*fliC*) of *P. plecoglossicida*. By comparing it with the wild-type strain (WT), we aim to elucidate the molecular mechanism through which bacterial flagella interfere with host energy metabolism, thereby advancing our understanding of flagellar biology.

## 2. Materials and Methods

### 2.1. Bacterial Strains and Culture Conditions

The *P. plecoglossicida* NZBD9 strain was stored at −80 °C in the laboratory. The *fliC* deletion mutant (Δ*fliC*) was constructed using a markerless knockout method as described by He [10]. Briefly, homology arms flanking the target *fliC* gene region were amplified using primers P1-P4 (Supplementary Table S1) and cloned into the suicide vector pK18mobSacB via seamless cloning. The resulting knockout plasmid was transformed into *E. coli* DH5α competent cells. Subsequently, the plasmid was extracted and electroporated into prepared *P. plecoglossicida* competent cells. The Δ*fliC* strain was selected using neomycin resistance and sucrose counter-selection. Successful deletion was confirmed by PCR using verification primers P5/P6 (Supplementary Table S1) and sequencing performed by Sangon Biotech (Shanghai, China), which verified the complete deletion of the *fliC* gene in strain NZBD9.

### 2.2. Transmission Electron Microscopy

The wild-type and Δ*fliC* strains were cultured overnight at 18 °C, then centrifuged at 4500 rpm for 8 min at 4 °C. The bacterial pellet was resuspended in sterile phosphate-buffered saline (PBS) to remove the culture medium, and this process was repeated twice. Using a pipette, 20 μL of the bacterial suspension was placed on a carbon-coated copper grid for 3 min, followed by removal of excess liquid with filter paper. A total of 2% phosphotungstic acid solution was applied to the grid for 1 min, excess liquid was removed with filter paper, and the grid was air-dried at room temperature. The sample was observed under a transmission electron microscope, and images were captured for analysis.

### 2.3. Determination of Growth Curve

The NZBD9 strain and ∆*fliC* strain of *P. plecoglossicida* were cultured in LB broth at 18 °C with shaking at 220 rpm/min overnight and then diluted with sterile LB broth to OD600 = 0.28 ± 0.01. Aliquots of 20 µL bacterial suspension and 180 µL LB broth were added to a 96-well plate. There were 12 replicates for each strain, and 200 µL of LB broth with 12 wells used for the control group. The OD value at 600 nm was measured at 18 °C with a multifunctional microtiter plate detector (every 30 min for 48 h).

### 2.4. Animal Experiments

Large yellow croakers were purchased from a farm in Ningde, China, with an average body weight of 150 ± 6.7 g. After acclimatization for 14 days in a recirculating seawater system at 18 ± 1 °C, the fish were used for infection experiments. During the acclimatization period, fish were fed once daily to satiation. Both bacterial strains were cultured overnight in LB broth at 18 °C, with shaking at 220 rpm. Cultures were centrifuged at 4500 rpm for 10 min, and the bacterial pellets were resuspended in sterile phosphate-buffered saline (PBS). This washing step was repeated twice to remove residual culture medium. The bacterial suspension was adjusted with PBS to an infection dose of 5 × 10^4^ CFU per fish. Each fish was intraperitoneally injected with 200 μL of the bacterial suspension or sterile PBS.

A total of 180 fish were utilized for the survival assay, with 60 fish per independent experiment, and the trial was repeated three times. For each experiment, 60 fish were randomly assigned and evenly distributed into three groups (*n* = 20 per group): wild-type infection group (WT group), Δ*fliC* infection group (Δ*fliC* group), and PBS injection group (control group). The WT and Δ*fliC* groups were administered the respective bacterial strains at a dose of 5 × 10^4^ CFU per fish, whereas the control group received an equivalent volume of PBS. Mortality was monitored and recorded daily, with deceased fish being immediately removed from the tank. The experimental period spanned 10 days.

Additionally, 270 fish were randomly allocated into three experimental sets (*n* = 90 per set), designated for microscope observation, metabolic parameter measurement, and high-throughput sequencing, respectively. Within each set, 90 fish were further evenly divided into three subgroups: the control group, WT group, and Δ*fliC* group, each receiving injections as previously described. Liver samples were collected from control and moribund fish in infected groups for subsequent experimental analyses (moribund fish were defined as those showing symptoms such as surfacing, and cessation of swimming with continued opercular movement after infection with the half-lethal dose), with a minimum of three biological replicates obtained per subgroup.

### 2.5. Microscope Observation

For histopathologic observation, part of the samples was dehydrated in a 70–100% ethanol gradient, then transparent in xylene, embedded in paraffin, and cut into 4 μm sections with a Leica microtome (Leica Biosystems, Nussloch, Germany). Sections were dewaxed, stained with hematoxylin, rinsed with ultrapure water, dehydrated through an ethanol gradient, and counterstained with 0.5% eosin solution. Another set of sections was dewaxed and stained using a PAS staining kit (Solarbio, Beijing, China) according to the manufacturer’s instructions. Briefly, sections were alternately stained with PAS solution C and PAS solution B for 15 min each, rinsed with pure water, stained with PAS solution A in the dark for 30 min, rinsed with pure water for 5 min, and finally dehydrated through a gradient series. Subsequently, the sections were transparent through xylene and sealed with neutral gum. After drying, the slides were observed and photographed using a Leica microscope. For vacuolated cell quantification, 10 randomly selected fields of view (FOVs) were examined to count vacuolated cells within each field. For PAS-staining grayscale analysis, the mean grayscale value of PAS staining was measured and statistically analyzed across 10 additional randomly selected FOVs.

For ultramicrostructure observation, the approach was followed Sun [13]. Briefly, 0.1 M phosphate buffer (pH 7.4) for 15 min, thrice; 50% ethanol for 15 min; 70% ethanol for 15 min; 80% ethanol for 15 min; 90% ethanol for 15 min; 95% ethanol for 15 min; 100% ethanol for 15 min, twice; acetone/epon-812 = 1:1 overnight; 60 °C 48 h; sliced; 2% uranyl acetate for 15 min; and lead citrate for 15 min, dried at room temperature overnight.

### 2.6. Metabolic Parameter Measurement

The following kits were used according to the manufacturers’ instructions to measure respective parameters in the livers of the three groups of large yellow croakers: Cell Mitochondrial Complex V (F0F1-ATPase/ATP Synthase) Activity Assay Kit (Elabscience, Wuhan, China); Amplex Red Triglyceride Assay Kit (Beyotime, Shanghai, China); Lipid Peroxidation Assay Kit (Nanjing Jiancheng, Nanjing, China); Lactate Dehydrogenase (LDH) Activity Assay Kit (Elabscience, Wuhan, China); GSH and GSSG Assay Kit (Beyotime, Shanghai, China); Enhanced NAD+/NADH Assay Kit with WST-8 (Beyotime, Shanghai, China); and Liver Glycogen Assay Kit (Nanjing Jiancheng, Nanjing, China). The parameters measured included ATP Synthase Complex V activity; LDH specific activity; lipid peroxide content; Triacylglyceride content; total GSH content; liver glycogen content; and the ratio of NAD+/NADH.

### 2.7. qRT-PCR Analysis for Transcription of Immune-Related Genes

The total RNA of each sample was extracted from the infected large yellow croaker tissues according to the manufacturer’s instructions of TransZol Up Kit (TransGen Biotech, Beijing, China). The cDNA was obtained using TransScript^®^ One-Step gDNA Removal cDNA Synthesis SuperMix (TransGen Biotech, Beijing, China). *β-actin* gene was used as the internal reference gene for this work. The mRNA relative expression level of target genes was calculated using 2^−ΔΔC^t method [14]. Primer sequences of genes used in this work are listed in Supplementary Table S1.

### 2.8. Proteomics Analysis

After adding protein lysis buffer, samples were homogenized three times, followed by 30 min of low-temperature sonication. Supernatants were collected by centrifugation, with protein concentration determined by BCA assay and analyzed via SDS-PAGE electrophoresis [15]. A total of 100 μg protein was reacted with 100 mM TEAB and 10 mM TCEP, followed by 40 min reaction with 40 mM iodoacetamide. Acetone-precipitated samples were centrifuged, dissolved, and digested. Peptides were vacuum concentrated [16]. Samples were redissolved in 0.1% TFA, desalted via HLB cartridges, vacuum concentrated, and finally quantified using a NanoDrop One UV spectrophotometer (Thermo, Waltham, MA, USA) [17].

Peptides were separated by Vanquish Neo LC system and analyzed on Orbitrap Astral MS, with data acquired using Thermo Xcalibur 4.7 [18,19]. DIA mode (100–1700 m/z) was used. Raw data were processed with Spectronaut™ 19 (large yellow croaker database) for bioinformatic analysis [20]. GO and KEGG pathway analyses were performed for functional clustering of differentially expressed proteins, and R language *t*-test was used to calculate the significance (*p* value) and fold change between groups [21,22].

### 2.9. Drawings and Statistical Analysis

Adobe Illustrator(version 2024) (San Jose, CA, USA) were used for drawings. The data were shown as means ± standard deviation (SD) and analyzed with two-way ANOVA, followed by Sidak’s multiple comparisons test using the analysis tool available in the Graphpad Prism software (version 8.0.1). *p* < 0.05 was considered as statistically significant. Details of the software and database used in this work are shown in Supplementary Table S2.

## 3. Results

### 3.1. fliC Contributed to the Pathogenesis of P. plecoglossicida

**Figure 1:**
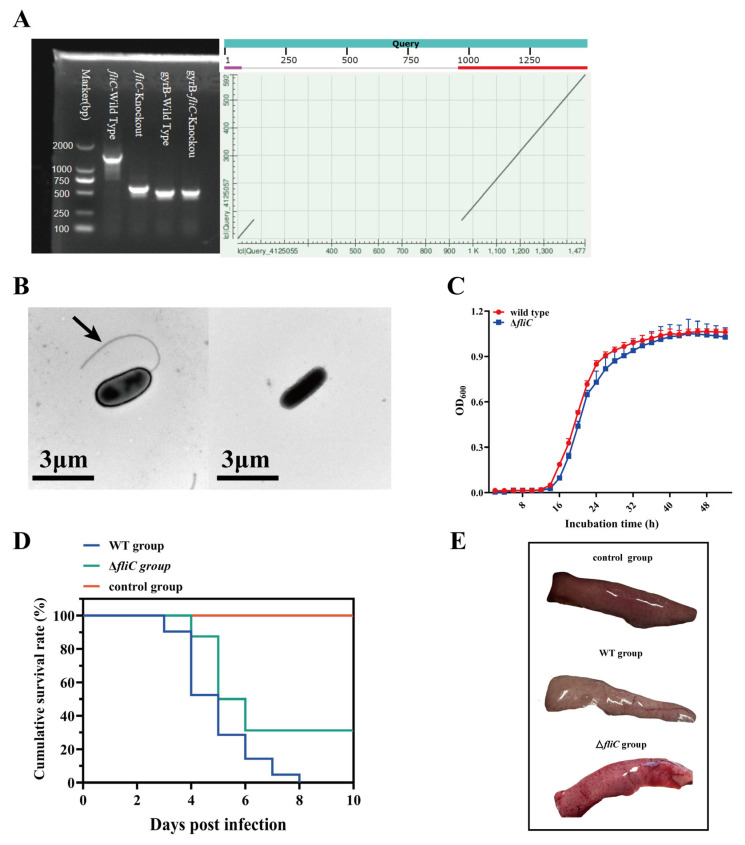
The *fliC* gene contributed to the pathogenesis of *P. plecoglossicida*. (**A**): Construction of *P. plecoglossicida* ∆*fliC* mutant strain. (**B**): Electron microscopy of WT and ∆*fliC;* arrows indicate the flagella of the WT strain. (**C**): *In vitro* growth kinetics of WT and ∆*fliC*. (**D**): Survival rates of large yellow croakers infected with WT or ∆*fliC*. (**E**): Livers of moribund large yellow croakers infected with WT or ∆*fliC*.

The *fliC* gene encodes the bacterial flagellar filament. PCR and sequencing results confirmed the successful deletion of *fliC* from the genome of the WT (Figure 1A); *gyrB* was used as the reference gene for PCR amplification to verify the successful construction of the ∆*fliC* mutant [23]. Transmission electron microscopy showed the absence of the flagella in the Δ*fliC* strain (Figure 1B). *In vitro*, the growth rate of the WT and Δ*fliC* showed no significant difference (Figure 1C), which indicated that deletion of the *fliC* did not impair the growth of *P. plecoglossicida*. Large yellow croakers were challenged with the WT or Δ*fliC* at a dose of 5 × 10^4^ colony-forming units (cfu)/fish. The mortality of the WT group occurred at 3 days post-infection (dpi), with a final survival rate of 0%, and the mortality of the Δ*fliC* group occurred at 4 dpi, with a final survival rate of 35%, and no mortality occurred in the control group (Figure 1D). The result indicated that *fliC* contributed to the pathogenesis of *P. plecoglossicida*. Compared to the control group, the livers of infected croakers were paler and friable; however, the WT group was more severe than the Δ*fliC* group (Figure 1E). These results demonstrated that the *fliC* is not only involved in the pathogenesis to large yellow croakers but also associated with the liver damage of large yellow croakers.

### 3.2. fliC Disrupted Energy Metabolism in Large Yellow Croaker Livers

**Figure 2:**
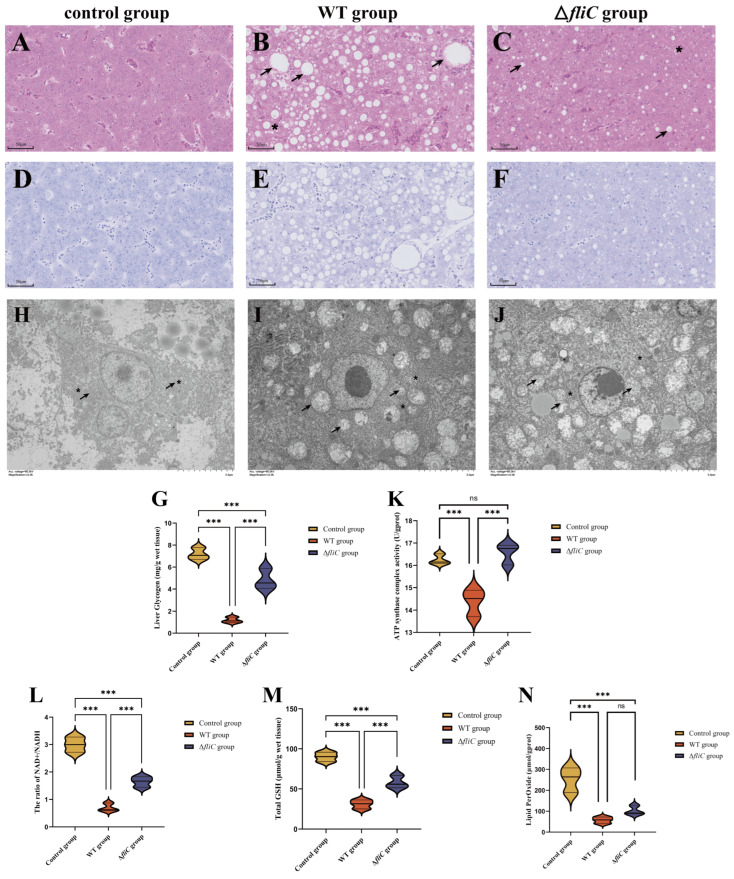
*fliC* involved in disruption of energy metabolism in large yellow croaker livers. (**A**–**F**): Hepatic histopathological features. (**A**–**C**): HE staining. Arrows indicate vacuolar degeneration cells; asterisk indicated infiltrating inflammatory cells. (**D**–**F**): PAS staining; (**A**,**D**): control group; (**B**,**E**): WT group; and (**C**,**F**): Δ*fliC* group. (**H**–**J**): Hepatic ultrastructural features of livers. (**H**): control group. (**I**): WT group. (**J**): Δ*fliC* group. Arrows indicate mitochondria, asterisk indicates endoplasmic reticulum. (**G**,**K**–**N**): livers of large yellow croaker’s liver glycogen (**G**), total liver ATP synthase complex activity (**K**), the ratio of NAD+/NADH (**L**), total liver GSH (**M**), and total liver lipid peroxide (**N**) were determined. ns: not significant. ***: *p* < 0.01.

The pathological results showed that hepatocytes in the control group were relatively well-organized without significant lesions (Figure 2A), whereas varying degrees of vacuolar degeneration and inflammatory cell infiltration were observed both in the WT (Figure 2B) and Δ*fliC* groups (Figure 2C). Notably, the Δ*fliC* group showed a 29.54% reduction in the vacuolated cell count compared to the WT (Supplementary Figure S1), indicating *fliC* involvement in the *P. plecoglossicida*-induced disruption of the hepatic energy metabolism. In addition, infection caused a reduction in liver glycogen. Compared with the control group, the Δ*fliC* group decreased by 32.70%, while the WT group was more severe, decreasing by 83.53% (Figure 2D–G). The results indicated that *fliC* participated in interfering with glucose metabolism. On the other hand, the mitochondria and endoplasmic reticulum were intact in the control group. In the WT group, the cristae of both endoplasmic reticulum and mitochondria disappeared (Figure 2I). In the Δ*fliC* group, the cristae of endoplasmic reticulum and mitochondria were fractured (Figure 2J). What is more, infection caused a decrease in mitochondrial complex V activity and the NAD+/NADH ratio, reduced glutathione levels, and caused an increase in lipid peroxides. However, the Δ*fliC* group was better than the WT group (Figure 2K–N). These results suggested that *fliC* contributed to *P. plecoglossicida*-induced energy metabolism dysregulation.

### 3.3. Proteomics Analysis of Large Yellow Croaker Livers

**Figure 3:**
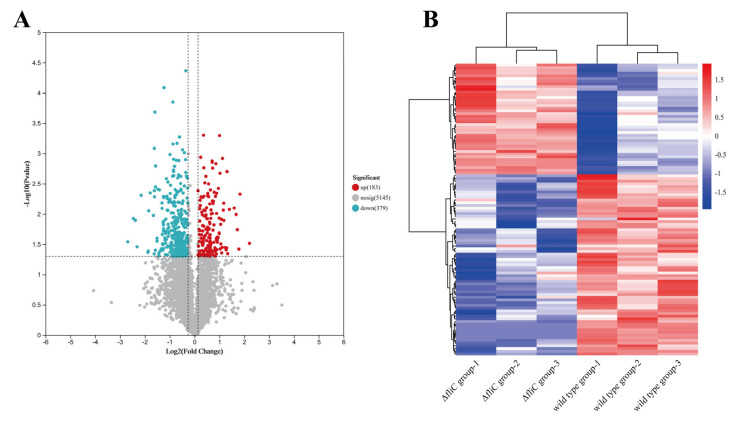
Analysis of differentially expressed proteins in livers of large yellow croakers infected with WT and Δ*fliC*. The volcano plot (**A**) and heatmap (**B**) of differentially expressed proteins in livers of large yellow croakers infected with the WT or Δ*fliC*.

To comprehensively characterize the *fliC*-mediated disruption of the energy metabolism, livers from large yellow croakers infected with WT or ∆*fliC* were examined. Compared to the WT group, the Δ*fliC* group had 562 differentially expressed proteins, among which 183 proteins were significantly upregulated, and 379 proteins were significantly downregulated (Figure 3A). All differentially expressed proteins (DEPs) were reproducible across biological replicates (Figure 3B).

**Figure 4:**
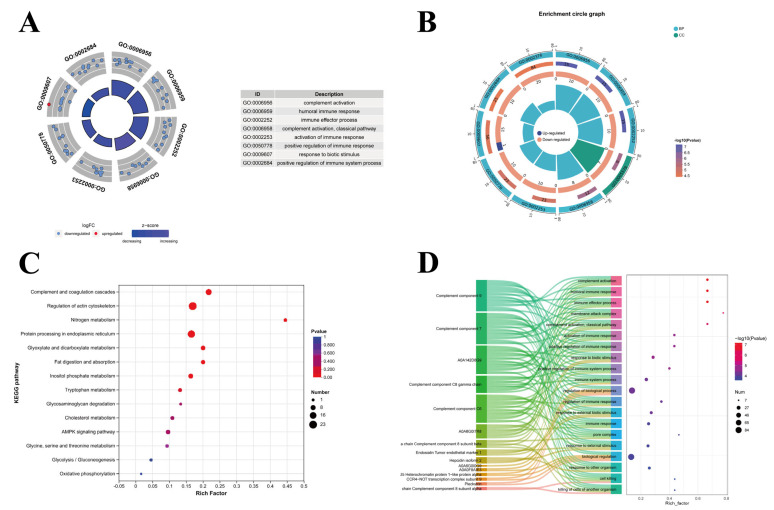
Comparative proteomics analysis between the livers of large yellow croakers infected with WT and Δ*fliC*. (**A**,**B**): GO enrichment analysis related to immune response. (**C**): KEGG pathway enrichment analysis for differentially expressed proteins. (**D**): Sankey diagram illustrating interactions of the complement component family with enriched pathways.

A GO enrichment analysis revealed that the following functions were most significantly affected by *fliC*: complement activation, humoral immune response, immune effector process, activation of immune response, positive regulation of immune response, response to biotic stimulus, and positive regulation of immune system process (Figure 4A). Red circles denote upregulated DEPs within the terms; blue circles denote downregulated DEPs. To further illustrate the GO enrichment levels, a multi-dimensional circle plot was generated based on the DEPs and GO enrichment results (Figure 4B). Notably, these GO terms were predominantly enriched in downregulated proteins, with the exception of “response to biotic stimulus” (GO:0009607), which contained one upregulated protein. This pattern suggested that *fliC* triggered an immune activation in large yellow croakers. Concurrently, the KEGG pathway analysis revealed the following pathways as most significantly impacted by *fliC*: oxidative phosphorylation, glycolysis/gluconeogenesis, glycine, serine and threonine metabolism, AMPK signaling pathway, cholesterol metabolism, glycosaminoglycan degradation, tryptophan metabolism, inositol phosphate metabolism, fat digestion and absorption, glyoxylate and dicarboxylate metabolism, protein processing in endoplasmic reticulum, nitrogen metabolism, regulation of actin cytoskeleton, and complement and coagulation cascades (Figure 4C). Unexpectedly, both the complement and coagulation cascades and central carbohydrate metabolism pathways were significantly enriched. Notably, the complement cascade also participated in immune-related pathways (Figure 4D). A KEGG analysis indicated that *fliC* actively contributed to the disruption of complement-mediated immunity and carbohydrate metabolism pathway regulation.

### 3.4. fliC May Trigger Immune and Metabolic Dysregulation Through Tlr5 Signaling

To dissect the mechanism of *fliC*-mediated dysregulation in carbohydrate metabolism pathways, we quantified the expression of pivotal regulatory genes governing these processes.

**Figure 5:**
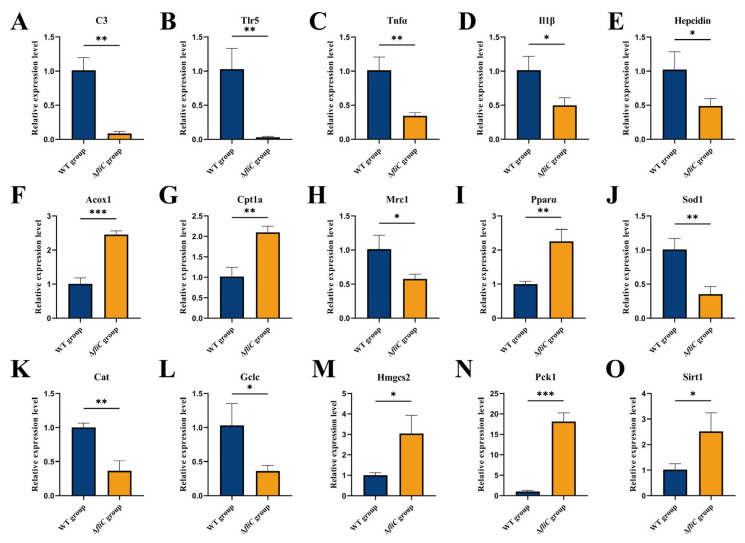
Relative expression levels of immunometabolic genes in the livers of large yellow croakers infected with WT and Δ*fliC*. (**A**) *C3* (complement component 3), (**B**) *Tlr5* (Toll-like receptor 5), (**C**) *Tnfα* (tumor necrosis factor-alpha), (**D**) *Il1β* (interleukin-1 beta), (**E**) *Hepcidin* (iron-regulatory antimicrobial peptide), (**F**) *Acox1* (acyl-CoA oxidase 1), (**G**) *Cpt1a* (carnitine palmitoyltransferase 1A), (**H**) *Mrc1* (macrophage mannose receptor 1), (**I**) *Pparα* (peroxisome proliferator-activated receptor alpha), (**J**) *Sod1* (superoxide dismutase 1), (**K**) *Cat* (catalase), (**L**) *Gclc* (glutamate–cysteine ligase catalytic subunit), (**M**) *Hmgcs2* (3-hydroxy-3-methylglutaryl-CoA synthase 2), (**N**) *Pck1* (phosphoenolpyruvate carboxykinase 1), and (**O**) *Sirt1* (sirtuin 1). All values are expressed as mean ± SD, *n* = 3. Significant differences between the WT and Δ*fliC* groups are indicated by asterisks: *, *p* < 0.1; **, *p* < 0.05; ***, *p* < 0.01.

**Figure 6:**
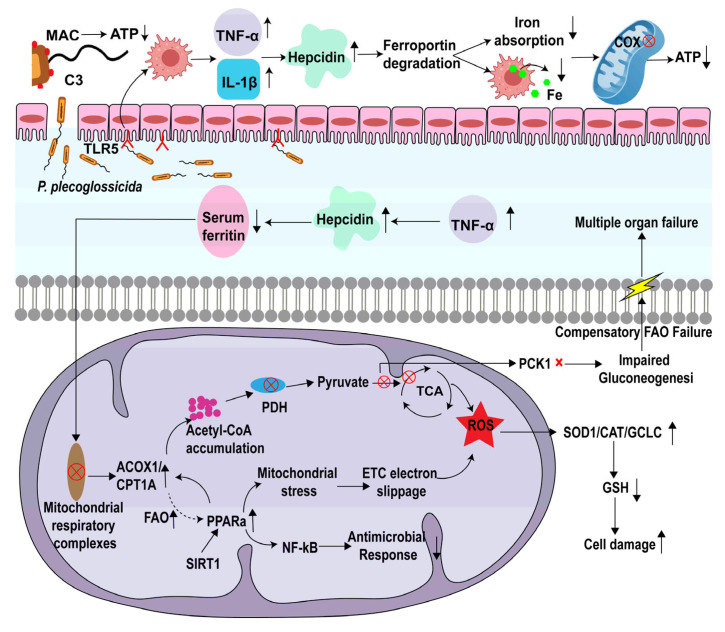
Putative schematic diagram illustrating *fliC*-triggered immune and metabolic dysregulation through Tlr5 signaling. The “↑” denotes upregulation of genes or proteins. while the cross symbol denotes the halting of synthesis. The “×” indicates down regulation of genes or proteins. The “⭙” indicates the metabolic pathway is blocked.

Additionally, to investigate the abovementioned immune responses and metabolic pathways following infection, we conducted a quantitative real-time PCR (qRT-PCR) analysis of 15 key genes involved in immune regulation, iron homeostasis, lipid metabolism, oxidative stress, and metabolic signaling. The gene patterns that were analyzed are *Tlr5*, *C3*, *Tnfα*, *Il1β*, *Hepcidin*, *Acox1*, *Cpt1a*, *Mrc1*, *Pparα*, *Sod1*, *Cat*, *Gclc*, *Hmgcs2*, *Pck1*, and *Sirt1* (Figure 5). The complement component *C3* was downregulated, indicating the suppression of the complement pathway. Similarly, pro-inflammatory cytokine genes *Tnfα* and *Il1β* were downregulated, alongside Tlr5, suggesting a subdued immune activation in the Δ*fliC* group. Conversely, genes involved in fatty acid β-oxidation and mitochondrial fatty acid transport—*Acox1*, *Cpt1a*, and *Pparα*—were significantly upregulated, indicating an enhanced lipid catabolism. Oxidative stress-related genes *Sod1* and *Cat*, as well as the glutathione synthesis enzyme *Gclc*, were downregulated, reflecting alterations in the redox balance. Furthermore, genes associated with gluconeogenesis (*Pck1*) and ketogenesis (*Hmgcs2*) were suppressed. Notably, the energy sensor *Sirt1*, which links immune regulation to metabolic pathways, was upregulated in the Δ*fliC* group. These results indicated that *fliC* may trigger a *Tnfα*/*Il1β* cytokine storm through tlr5, thereby interfering with carbohydrate metabolism (Figure 6).

## 4. Discussion

Upon pathogenic challenge, the host initiates metabolic reprogramming to fuel immune responses [24]. Conversely, to prevent the pathogen exploitation of nutrients, the host employs nutritional immunity by sequestering or expelling energy substrates [25]. Regardless, infection inevitably disrupts metabolic homeostasis and drives its restructuring. *P. plecoglossicida* induces a cytokine storm in large yellow croakers [11]. The flagella serve as one of the inflammatory triggers. For instance, *Salmonella Typhi* flagella activated the host TLR5-mediated innate immunity, and their deletion not only alleviated inflammation or downregulated inflammatory markers such as TNF-α/IL-1β [26] but also reduced virulence. In the case of *P. plecoglossicida*, *fliC* deletion led to attenuated virulence (Figure 1D) and alleviated inflammation (Figure 5).

The activation of inflammation by *fliC* may be driven by Tlr5, a member of the Toll-like receptor family that specifically recognizes the flagella and transmits signals to activate downstream immune responses [27]. In severe cases, this TLR5-driven immune response may become dysregulated, leading to tissue damage [28]. This excessive inflammatory response also consumes substantial amounts of energy, thereby imposing an increased burden on mitochondria and causing mitochondrial injury [29]. More critically, mitochondria operating at full capacity generate vast quantities of ROS. When the sheer volume of ROS surpasses the neutralization capacity of limited antioxidants such as glutathione (GSH), indiscriminate attacks on host cellular components occur, further exacerbating inflammation [30]. Concurrently, the elevated expression of Sod1, Cat, and Gclc signifies the formation of an antioxidant enzymatic consortium comprising superoxide dismutase (SOD), catalase (CAT), and glutamate–cysteine ligase (GCL) (Figure 6). This consortium not only directly contributes to mitigating inflammation but also buys crucial time for the large yellow croaker to mount more effective countermeasures.

Notably, an important consideration arising from our findings is whether the attenuated pathogenicity of the Δ*fliC* strain and its reduced impact on host liver metabolism are primarily due to the loss of flagellum-mediated motility or the absence of *fliC*-specific TLR5 signaling. It is well-established that flagellar motility is critical for bacterial colonization, host invasion, and biofilm formation [1,2]. Therefore, the impaired motility of the Δ*fliC* mutant likely contributes to its reduced virulence by diminishing its ability to effectively reach and colonize host tissues. The significant downregulation of the TLR5/NF-κB pathway (*Tlr5*, *Tnfα*, and *Il1β*), the altered regulation of complement cascades, and the concomitant mitigation of metabolic dysregulation in the Δ*fliC* group collectively suggest that the immunomodulatory role of the *fliC* protein itself is a major driver of the observed immunometabolic reprogramming. Although the two mechanisms—loss of motility and loss of TLR5 signaling—are not mutually exclusive and may operate synergistically, our results suggest that the hyperactivation of pro-inflammatory immunity via the *fliC*-TLR5 interaction may exacerbate metabolic perturbations independent of the consequences of reduced bacterial motility. Our study underscores that the flagellum is not only a motility organelle but also a key immunometabolic regulator in *P. plecoglossicida* pathogenesis.

An activated immune state is highly energy demanding [31]. Here, we observed that the hepatic energy metabolism in the ∆*fliC* group was significantly healthier compared to the WT group (Figure 2D–F). This metabolic shift was not solely attributable to mitochondrial damage but also potentially linked to modulated inflammation. For instance, the TLR5-TNFα signaling axis indirectly regulates insulin receptor substrates (IRSs); their downregulated expression suggested the potential impairment of the insulin signaling pathway (Figure 2G) [32]. Concurrently, the activation of PCK1 (phosphoenolpyruvate carboxykinase) contributed to maintaining blood glucose levels (Figure 5N) [33]. Furthermore, the upregulation of PPARα, ACOX1, and CPT1A promoted the accumulation of acetyl-CoA derived from enhanced β-oxidation (Figure 5F,G,I) [34]. Excess acetyl-CoA exerted feedback inhibition on pyruvate dehydrogenase (PDH), thereby suppressing both glycolysis and the TCA cycle [35]. Through this coordinated multisystem interplay between immunity and metabolism, the large yellow croaker exhibited enhanced resistance to the ∆*fliC* infection.

## 5. Conclusions

In conclusion, our findings demonstrated that the *fliC*-encoded flagellin mediates *P. plecoglossicida*’s disruption of the core pathways governing inflammatory immunity and glucose metabolism in the large yellow croaker. This disruption involved pathological processes including glycogen depletion and mitochondrial damage. Deletion of the *fliC* gene resulted in a significant 30% reduction in bacterial virulence. Furthermore, these results elucidate the mechanisms by which the *fliC* mediated bacterial pathogenicity and provide novel insights into the regulatory networks linking infection-induced immune responses and metabolic reprogramming.

## Data Availability

The original contributions presented in this study are included in the article/supplementary material. Further inquiries can be directed to the corresponding author.

## Supplementary Materials

The following supporting information can be downloaded at: https://www.mdpi.com/article/10.3390/antiox14101189/s1, Figure S1: Quantitative differences in vacuolated cells, ***, *p* < 0.01; Table S1: The sequence of primers for qRT-PCR; and Table S2: Software and database information.
