# Supplementary material for: fliC Mediates Pseudomonas plecoglossicida’s Hijack of Inflammatory Immunity and Glucose Metabolism in the Large Yellow Croaker
Source: Antioxidants (Basel). 2025 Sep 28;14(10):1189. doi: 10.3390/antiox14101189 (PMC12561762; doi:10.3390/antiox14101189)
Supplement: Supplementary file 1 [file antioxidants-14-01189-s001.zip › antioxidants-3850220-supplementary.pdf]

Supplementary material

Supplementary Table S1. The sequence of primers for qRT-PCR

Primer	Sequence (5’-3’)
gyrB-F	TGCTGAAGGACGAGCGTTCG
gyrB-R	GAATATTGTTGGTGAAGCAGGCTA
P1	CAGGTCGACTCTAGAGGATCCTGAGCTTTCGAGGGTACAGCG
P2	TAACAAGGGACACACCTCACGCAGCCCATACGCCCT
P3	GTGAGGTGTGTCCCTTGTTAGTCAATC
P4	TATGACCATGATTACGAATTCGTCAAGCCGATGAAGTCATCG
P5	AGGAGCAAGTAGTGGTAGA
P6	AGTCAGCGTATTCGTTCAG
β-actin-F	GGCTACTCCTTCACCACCACAG
β-actin-R	TCTGGGCAACGGAACCTCT
C3-F	CTTATATGGCACAGGAGACA
C3 -R	CCGATTCTGACAGTGGTAG
Tlr5-F	GTGCCAATCCAACAACATT
Tlr5-R	ATAGCAAGCGTCCACATAC
Tnfa-F	ACTGCCGAGAAAACAGATCCTC
Tnfa-R	TTCACCGTTCTTCCACTCCAG
Il1β-F	GCTGAGAACCGCAAAGTTCAC
Il1β-R	CTCCAGATGCAAGGTTGGGT
Hepcidin-F	ACCATCAGACAGCAGGAA
Hepcidin-R	AGAGCACCACAATATCTTCA
Mrc1-F	TCATCCATCCATCCATTCAG
Mrc1-R	GGCATCCACATCTACTTCTT
Pparα-F	ATTCTCCGACTGATGACATT
Pparα-R	AGCACATTCCATTCACATTC
Acox1-F	CTTAACTTCACCTGTCTCAC
Acox1-R	GAACTCTCCGATGCTCTG
Cpt1a-F	TAAGATGCTACCTGCCTAAC
Cpt1a-R	GTCGTTGGAACTACTGTGA
Sod1-F	CTTATCGTCACCAGGATTCT
Sod1-R	GCCATTACAGCATCAGTTAG
Cat-F	AAGATAATGTCACGCAGGTA
Cat-R	GATAGCCAGTCAGCAAGAA
Pck1-F	GCAAGCAAGGAGTCAGAA
Pck1-R	CAGAGATGTCAGCGTGTT
Gclc-F	AGCAGAGACAGGTAGCAT
Gclc-R	ATGAATGATGAACGGAGACT
Acaa2-F	CAGGTTACTACACAGCAGAA
Acaa2-R	TGAAGACAGGAGCCAGTT
Sirt1-F	TCCGAGGACCATTCTGAG
*Sirt1*R	GGAGGTTCTGAGATGTTGAT

Supplementary Table S2. Software and database information

Soft/Database	Version	Analysis	Source
JASPAR	Version 2024	Functional annotation of whole proteome	https://jaspar.elixir.no/
ngloc	-	Functional annotation of whole proteome	https://pubmed.ncbi.nlm.nih.gov/17472741/
HMMER	3.1b2	Functional annotation of whole proteome	https://www.ebi.ac.uk/Tools/hmmer
DIAMOND	Version 2.1.9	Functional annotation of whole proteome	https://github.com/bbuchfink/diamond
signalp	Version 6.0	Functional annotation of whole proteome	https://services.healthtech.dtu.dk/services/SignalP-6.0/
R	-	Differential protein analysis	https://www.r-project.org/
caret	Version 6.0-94	Data Modeling: Classification and Regression	https://cran.r-project.org/web/packages/caret/index.html
Uniprot	Version 2023.11	Functional annotation of whole proteome	ftp://ftp.uniprot.org/pub/databases/uniprot/current_release
Goatools	Version 1.4.4	GO enrichment analysis	https://pypi.org/project/goatools/
Python	-	KEGG enrichment analysis	https://www.python.org/
Graphpad prism	Version 8.0.1	Statistic analysis of data	http://s.whhaox.cn/Graphpadprism/1.html
Adobe Illustrator	Version2020	Drawing	https://www.adobe.com/cn/products/illustrator.html

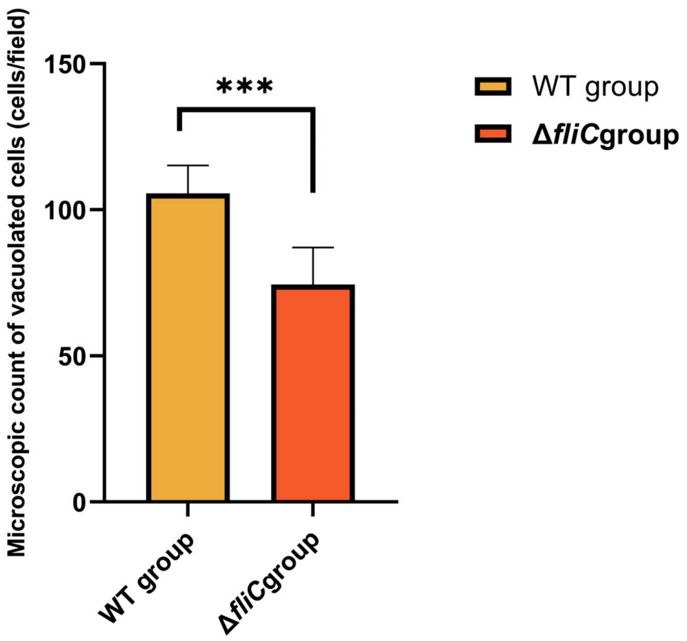

Supplementary figure S1 Quantitative differences in vacuolated cells, ***, P < 0.01
